# In *Aspergillus nidulans* the Suppressors *suaA* and *suaC* Code for Release Factors eRF1 and eRF3 and *suaD* Codes for a Glutamine tRNA

**DOI:** 10.1534/g3.114.010702

**Published:** 2014-04-09

**Authors:** Wen Liu, Laura Mellado, Eduardo A. Espeso, Heather M. Sealy-Lewis

**Affiliations:** * Department of Biological, Biomedical and Environmental Sciences, University of Hull, Hull HU6 7RX, United Kingdom; † Department of Cellular and Molecular Biology, Centro de Investigaciones Biológicas (CSIC), Ramiro de Maeztu, 9, 28040 Madrid, Spain

**Keywords:** suppression of nonsense mutations in *A. nidulans*, *suaD* codes for a glutamine tRNA, *suaA* codes for eRF1, *suaC* codes for eRF3

## Abstract

In *Aspergillus nidulans*, after extensive mutagenesis, a collection of mutants was obtained and four suppressor loci were identified genetically that could suppress mutations in putative chain termination mutations in different genes. Suppressor mutations in *suaB* and *suaD* have a similar restricted spectrum of suppression and *suaB111* was previously shown to be an alteration in the anticodon of a gln tRNA. We have shown that like *suaB*, a *suaD* suppressor has a mutation in the anticodon of another gln tRNA allowing suppression of UAG mutations. Mutations in *suaA* and *suaC* had a broad spectrum of suppression. Four *suaA* mutations result in alterations in the coding region of the eukaryotic release factor, eRF1, and another *suaA* mutation has a mutation in the upstream region of eRF1 that prevents splicing of the first intron within the 5′UTR. Epitope tagging of eRF1 in this mutant results in 20% of the level of eRF1 compared to the wild-type. Two mutations in *suaC* result in alterations in the eukaryotic release factor, eRF3. This is the first description in *Aspergillus nidulans* of an alteration in eRF3 leading to suppression of chain termination mutations.

Isolation of genetic suppressor mutations of chain termination mutations (and anti-suppressors) has proved to be a very powerful tool for identifying components of the translation machinery in a range of model eukaryotic organisms including *Saccharomyces cerevisiae*, *Schizosaccharomyces pombe* (Hawthorne and Leupold 1974), *Podospora anserina* (Coppin-Raynal and Dequard-Chablat 1988), *Caenorhabditis elegans* (Hodgkin 2005), and *Aspergillus nidulans* (Martinelli 1994). The early genetic screens made predictions about the possible function of the suppressor genes from phenotypic observations and, much later, these predictions have been confirmed by the molecular data; however, to date this has not been accomplished with *Aspergillus nidulans*. The mechanism of chain termination is still poorly understood, and it is of interest not only from a fundamental scientific standpoint as it is ubiquitous in all cells, but also from a practical point of view because therapies are being developed to allow read-through of premature chain termination mutations in disease genes (Bidou *et al.* 2012; Bordeira-Carrico *et al.* 2012), and vector selection systems are being developed that have a nonsense mutation in the recipient strain and a suppressor mutation in the vector rather than using an antibiotic resistance gene for selection (Oliveira and Mairhofer 2013). In all the organisms where chain termination suppressors have been isolated and characterized at the molecular level, the release factors eRF1 and eRF3 have been found to be essential for polypeptide chain termination. Mutations in either of these proteins can lead to read-through of chain termination mutations (Gagny and Silar 1998; Inge-Vechtomov *et al.* 2003), but the release factors have other roles in cell organization and cell cycle progression, and characterization of the suppressors from a range of organisms may give important insights into these additional functions (Inge-Vechtomov *et al.* 2003).

In *Aspergillus nidulans*, four suppressor loci, *suaA*, *suaB*, *suaC*, and *suaD* (*sua*=suppressor in *Aspergillus*) were isolated by co-reversion of mutations in unrelated genes (Roberts *et al.* 1979). These suppressors were allele-specific and gene-unspecific and were thought to act on nonsense mutations because of the lack of any residual function in the gene products of the suppressible mutant strains. Subsequent work extended the number of co-suppressible alleles, but the number of new suppressor loci was limited and these have not been characterized further (Al Taho *et al.* 1984; Martinelli *et al.* 1984; Sealy-Lewis 1987). The suppressors in the *suaB* and *suaD* loci had a restricted pattern of suppression and were semi-dominant to the wild-type suppressor allele, whereas mutations in *suaC* and *suaD* had a wider spectrum of suppression and were recessive or semi-dominant depending on the suppressible allele being studied. The *suaA* and *suaC* alleles also had a number of pleiotropic alterations, like the omnipotent suppressors in *Saccharomyces cerevisiae*, that were able to suppress all three classes of chain termination mutation. These included cold sensitivity, slow growth in conditions not requiring the action of the suppressor, poor conidial viability, lowered fertility, and increased sensitivity to aminoglycoside antibiotics (Martinelli 1994). The behavior of the two classes of suppressor led to the hypothesis that the *suaB* and *suaD* mutations were acting by alterations in tRNA whereas the *suaA* and *suaC* mutations were acting by alterations in ribosomal proteins or release factors. The properties of the *suaA* and *suaC* suppressor strains have similarities to the *SUP45* and *SUP35* omnipotent suppressor strains in *Saccharomyces cerevisiae* and the *su2* and *su1* suppressors in *Podospora anserina* that have been shown to code for the release factors eRF1 and eRF3, respectively (Frolova *et al.* 1994; Stansfield *et al.* 1995; Gagny and Silar 1998). The *suaB111* mutation was identified as a G-to-A alteration in the anticodon CUG of a glutamine tRNA leading to recognition of UAG as a sense codon (Espeso *et al.* 2005), but the mechanism of action of the other suppressors has remained unidentified. In this article, we identify functions for *suaA*, *suaC* (eRF1 and eRF3), and *suaD* (glutamine tRNA).

## Materials and Methods

### *A. nidulans* strains, media, growth conditions, and manipulations

*Aspergillus* media and growth conditions were as described by Cove (1966). The scoring of the suppressible alleles in *alX*, *sB* and *niaD*, are described by Sealy-Lewis (1987). Genetic techniques were as described by Clutterbuck (1974). The strains used are listed in Supporting Information, Table S1. Gene symbols are as previously described (Clutterbuck 1993, 1997) and the characterized sequence changes of suppressible alleles are listed in Table 1.

**Table 1:** Alleles with known chain termination sequence changes

Mutant Allele	Nucleotide Change	Amino Acid Affected	Amino Acid Change	Reference
*alX4**^a^*	CAG to TAG	Gln 409	Gln to TAG	This study
*alcR125**^b^*	TGG to TAG	Trp 149	Trp to TAG	Dr. B. Felenbok, personal communication
*areA600*	TCG to TAG	Ser 646	Ser to TAG	Kudla *et al.* (1990) and Langdon *et al.* (1995)
*areA601*	AAA to TAA	Lys 206	Lys to TAA	Prof. H. N. Arst, personal communication
*aldA67*	UGG to UGA	Trp 131	Trp to TGA	Flipphi *et al.* (2001)
*acuH13*	CAG to TAG	Gln 134	Gln to TAA	Martinez *et al.* (2007)
*acuH31*	CAG to TAG	Gln 26	Gln to TAG	Martinez *et al.* (2007)
*acuH20*	TGG to TAG	Trp 254	Trp to TAG	Martinez *et al.* (2007)
*ngA1*	TTA to TGA	Leu 269	Leu to TGA	Han *et al.* (2005)
*palC143*	TTA to TGA	Leu 223	Leu to TGA	Tilburn *et al.* (2005)
*palF15*	TTA to TGA	Leu 189	Leu to TGA	Herranz *et al.* (2005)
*palB7*	GGA to TGA	Gly 791	Gly to TGA	Peñas *et al.* (2007)
*palB513*	TTA to TGA	Leu 552	Leu to TGA	Peñas *et al.* (2007)
*brlA23*	GAG to TAG	Gln 317	Gln to TAG	Griffith *et al.* (1999)
*brlA17*	GAA to TAA	Glu 118	Glu to TAA	Griffith *et al.* (1999)
*brlA19*	TAC to TAA	Tyr 395	Tyr to TAA	Griffith *et al.* (1999)
*brlA4*	CAA to TAA	Gln 334	Gln to TAA	Griffith *et al.* (1999)

a *alX4* is the gene for allantoinase, AN4603, C1218T.

b *alcR125* codes for the transcription factor AN8978, G506A to stop. Although all the mutants listed have chain termination mutations within the coding region, not all the mutants have been shown to be suppressible (see text).

### Molecular techniques

Standard molecular techniques are described by Sambrook *et al.* (1989). *Aspergillus* genomic DNA was prepared as described by Jones and Sealy-Lewis (1989). DNA was extracted from the strains [H3, H103, H44, H44(27), H44(23), H44(32), H7, H7 rev16] and PCR primers (Table S2) were designed to amplify overlapping sections of the entire coding region of the genes. The same primers were used for sequencing the PCR products. The coding region of both genes was sequenced on both strands. Where a change was identified compared with the sequence in the database, the wild-type was sequenced in that region to confirm the change. RT-PCR was performed using the GE Healthcare Illustra Ready-to-Go RT-PCR beads. The PCR products for eRF1 and the tubulin controls were standardly run on 1.3% TAE agarose gels or 5% TBE polyacrylamide gels. Crystal structure prediction of eRF1 and eRF3 was achieved through EsyPred3D Web server (http://www.fundp.ac.be/sciences/biologie/urbm/bioinfo/esypred/) and Swiss-model (http://swissmodel.expasy.org/).

### Transformation experiments, protein analyses, and cell imaging

**Figure 1:**
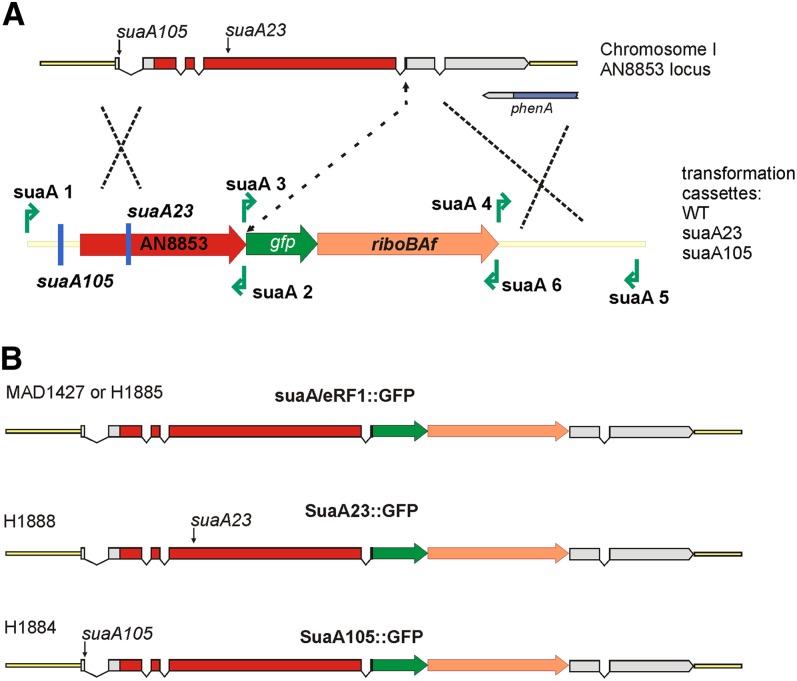
Generation of recombinant strains expressing GFP-tagged versions of SuaA. The cartoon (A) denotes the eRF1 locus (AN8853 on chromosome 1) indicating the position of the introns. The arrows show the positions of the single base changes for *suaA105* (in the splice site for the upstream ORF) and *suaA23* (in the coding region of the third exon). The cartoon below shows the DNA cassettes generated by fusion PCR used in transformation (constructs were either wild-type or contained the *suaA23* or *suaA105* mutation) together with the primer pairs that were used (green arrows). The cartoons in (B) show the strains transformed and the resultant status of the gene after recombination into the homologous eRF1 locus; on the top line the strains transformed with the wild-type *suaA*^+^ construct, MAD1427 (*pyrG89*, *pabaA1*; *argB2*; *ΔnkuA*::*argB*; *riboB2*) or H1885(*yA2 pantoB100*; *sB43*; *aldA67 riboB2*); on the second line: the *suaA23* construct transformed into H1888 (*pabaA1; yA2 pantoB100*; *alX4*; *riboB2*; *suaA23*); on the third line: the *suaA105* construct transformed into H1884 (*alX4 suaA105*; *riboB2*).

Transformation of *Aspergillus* was performed as described by Tilburn *et al.* (2005). Strains expressing GFP C-terminally tagged fusions of wild-type or mutant SuaA protein were generated as described by Yang *et al.* (2004). Transformation DNA cassettes were obtained by fusion PCR procedures of three fragments comprising, in the following order, the 5′-UTR/*suaACDS*, *gfp/riboB^Af^* and 3′-UTR region of *suaA*, as described in Figure 1. Strains MAD1427 and H1885 were used to generate strains expressing SuaA-GFP, and H1888 and H1884 were used to express SuaA105-GFP and SuaA23-GFP proteins, respectively.

The *suaA23* mutation was re-introduced into the suppressible strain H44 by transformation. The fragment containing the *suaA23* allele was PCR-amplified using gDNA from H44(23) as the template and primer pairs suaA1 and suaA2. Transformants were positively selected on glucose minimal medium containing allantoin 10 mg/100 ml (0.6 mM) as a nitrogen source and further purified to homokaryosis, and the presence of the *suaA23* mutation in several transformants was verified by sequencing.

Protein extraction followed the alkaline lysis extraction procedure (Hervás-Aguilar and Peñalva 2010). For Western blotting, usually 10 μl of total protein extracts were electrophoresed in 10% SDS-polyacrylamide gels and subsequently transferred to nitrocellulose filters using TransBlot TurboTransfer System (Bio-Rad). GFP tagged proteins were detected using mouse anti-GFP (1/5000; Roche). Actin, detected with mouse anti-γ actin antibody (1/50,000; ICN Biomedicals), was used as loading control. Peroxidase-conjugated goat anti-mouse IgG immunoglobulin (Jackson ImmunoResearch Laboratories) at 1/4000 was used as a secondary antibody. Peroxidase activity was detected with the Amersham Biosciences ECL kit.

For all microscopy experiments, supplemented watch minimal medium was inoculated with conidia, and cells (mostly germlings) were observed after incubation at 25° for 16 hr (Peñalva 2005) using uncoated glass-bottom dishes (MatTek Corporation, Ashland, MA). Fluorescence images were acquired with an upright Eclipse 80i microscope (Nikon, Melville, NY) equipped with Brightline GFP-3035B (Semrock, Rochester, NY), a 100-W mercury lamp epifluorescence module, Uniblitz (Rochester, NY) external shutter, a 60× 1.40–numerical aperture (NA) plan apochromat objective, and an ORCA ERG camera (Hamamatsu, Bridgewater, NJ).

## Results and Discussion

### *suaD* codes for a glutamine tRNA

The *suaD* gene maps on linkage group VII and is linked to *alcA/R*. The suppressors, *suaD103* and *suaD108*, have identical specificity to the *suaB111* suppressor, and this was found to be an alteration in a gln tRNA (Sealy-Lewis 1987; Espeso *et al.* 2005). Analysis of the annotated database revealed a single tRNA on the left arm of chromosome VII distal to *alcR* on contig 1.168, which was a gln tRNA. Sequencing of the gln tRNA for *suaD103* and *suaD108* revealed the same nucleotide change in both mutants, leading to an anticodon change so that the UAG codon could be recognized as a sense codon. Thus, both the *suaB* and *suaD* suppressor mutations are in glutamine tRNAs (Table 2). Consistent with this, *suaB111* and *suaD103/108* suppress *alX4*, which contains a premature UAG stop codon, although the *alcR125* and *areA600* mutations also contain UAG stop codons but are not suppressed (Sealy-Lewis 1987). The insertion of glutamine would restore the wild-type amino acid to allantoinase (Alx), but for the other two mutated proteins there would be a substitution of glutamine for tryptophan in AlcR and glutamine for serine in AreA600, which are presumably non-functional. There are 174 tRNAs annotated (Iriarte *et al.* 2012), and of these seven are glutamine tRNAs. There are two glutamine tRNAs that recognize the codon CAA and five that recognize the preferred codon CAG (Lloyd and Sharp 1991; Iriarte *et al.* 2012). The suppressor tRNAs that have been selected independently (*suaB111*, *suaD103*, *and suaD108*) are all in tRNAs that recognize CAG codons.

**Table 2:** Sequence changes in suppressors

Gene	Gene Product	Nucleotide Change	Gene Product Alteration
	*A. nidulans* Accession Number		
*suaB111**^a^*	gln tRNA AN9659	contig 161 (CTG to CTA)	Anticodon change
			recognizes UAG
*suaD103*	gln tRNA AN9669	contig 168 (CTG to CTA)	Anticodon change
*suaD108*	gln tRNA AN9669	contig 168 (CTG to CTA)	Anticodon change recognizes UAG
*suaA105*	eRF1 AN8853	−209 G to A	Alteration in upstream region
*suaA101*	eRF1 AN8853	(TCC to TAC) C104A	S35Y, N113K
		(AAC to AAG) C452G	
*suaA23*	eRF1 AN8853	(AAC to ATC) A505T	N131I
*suaA32*	eRF1 AN8853	(AAA to AAC) A656C	K181N
*suaA27*	eRF1 AN8853	(ATT to TCT) A978T.T979C	I289S
*suaC109*	eRF3 AN2080	(TAT to AAT) T1186A	Y396N
*suaC109*	eRF3 AN2080	(TAT to AAT) T1186A	Y396N, N415H
*suaC500*	eRF1 AN8853	(AAC to CAC) A1243C	E117K
*snpA6**^b^*	eRF1 ANIA_08853*^d^*	(GAG to AAG) G462A	G265S
*suaE7**^c^*		(GGT TOAGT) G906A	

a Espeso *et al.* (2005).

b Han *et al.* (2005).

c Martinez *et al.* (2007).

d ANIA_O8853 is the prefix given to the *suaE7* mutation (aspergillusgenome.org).

### *suaA* codes for the release factor eRF1

**Figure 2:**
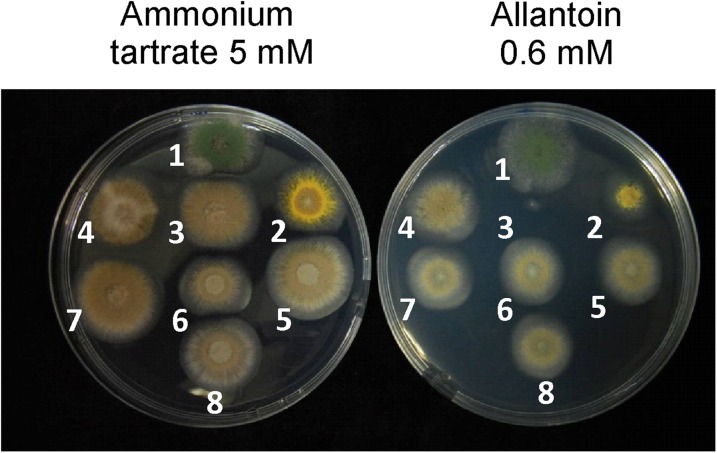
Growth testing of strains on 1% glucose minimal medium with 5 mM ammonium tartrate (left) or 0.6 mM allantoin as nitrogen source (right). The strains are control strains (only relevant genotype shown). 1. Wild-type; *alX*+ *suaA*^+^ (MAD2733). 2. *alX4 suaA105*. 3. *alX4 suaA*^+^. 4. *alX4 suaA23*. Individual transformants: 4, 5, 6, 7 of H44 (*alX4* containing strain transformed with a fragment containing the *suaA23* mutation; see *Materials and Methods*).

The *suaA* mutations were closely linked to each other and were shown not to recombine in large numbers of progeny, making it likely that they were all mutations within the same gene (Sealy-Lewis 1987). *suaA* mapped on chromosome III and was closely linked to *phenA* in the region of eRF1. *suaA101*, *suaA27*, *suaA32*, and *suaA23* all showed nucleotide changes in the coding region of eRF1 (ANIA 8853 version 5) (Table 2, Figure 1) compared to the wild-type sequence and the likelihood of there being a mutation in *suaA* in independent isolates if this is not the gene responsible for the phenotype must be very small. To confirm that a *suaA* mutation is responsible for the suppression, strain H44 (*pabaA1*; *alX4*; *alcR125*; *niaD500 fwA1*) was transformed with a fragment containing *suaA23* and allantoin-utilizing transformants were selected. The transformants were also suppressed for the *alcR125* and *niaD500* mutations showing suppressed growth on ethanol medium and nitrate medium, respectively (Figure 2).

**Figure 3:**
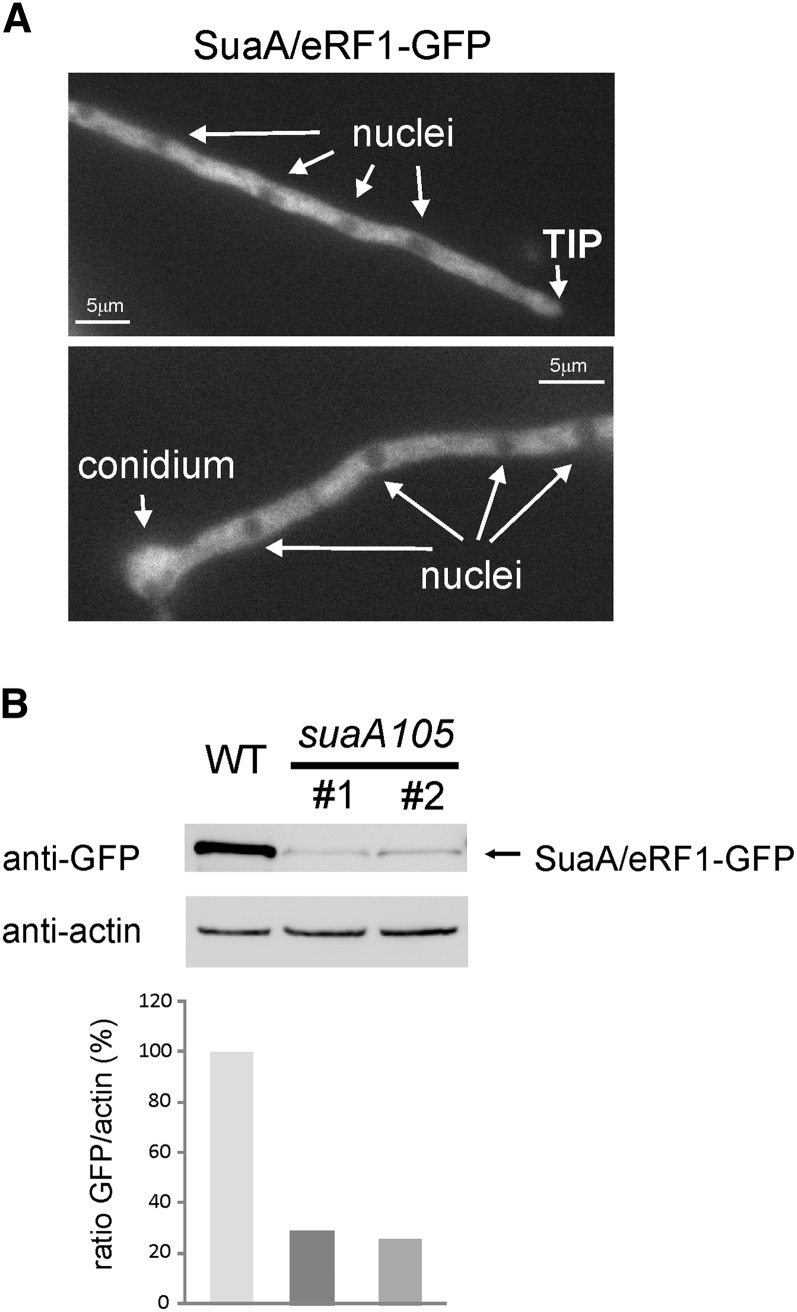
Localization and expression levels of SuaA/eRF1-GFP fusion. (A) Fluorescence images of cells of a transformant expressing SuaA-GFP fusion (H1885 transformed with the *suaA*::*gfp* construct; see Figure 1, MAD4903). (B) Western blot showing levels of SuaA-GFP fusion in total protein extracts of two transformants of *suaA105*::*gfp* (transformant 1 is MAD4904) compared with a *suaA^+^*::*gfp* transformant (MAD4903). Graph shows relative intensity of SuaA-GFP detection bands compared to actin levels.

Viable gene replacements of wild-type *suaA* with *suaA^+^* tagged with GFP (in strain H1885: *yA2 pantoB100*; *riboB2*; *alX4*; *sB43*; *aldA67*) were obtained that expressed the SuaA-GFP fusion showing that the GFP tag does not interfere with the function of eRF1 in chain termination. SuaA-GFP showed a preferential cytoplasmic localization where its activity is expected for translation termination (Figure 3A). Interestingly, nuclei were not fluorescent, suggesting the presence of a nuclear-export system acting on SuaA to ensure exclusion of the translational machinery from the nucleoplasm in this fungus.

**Figure 4:**
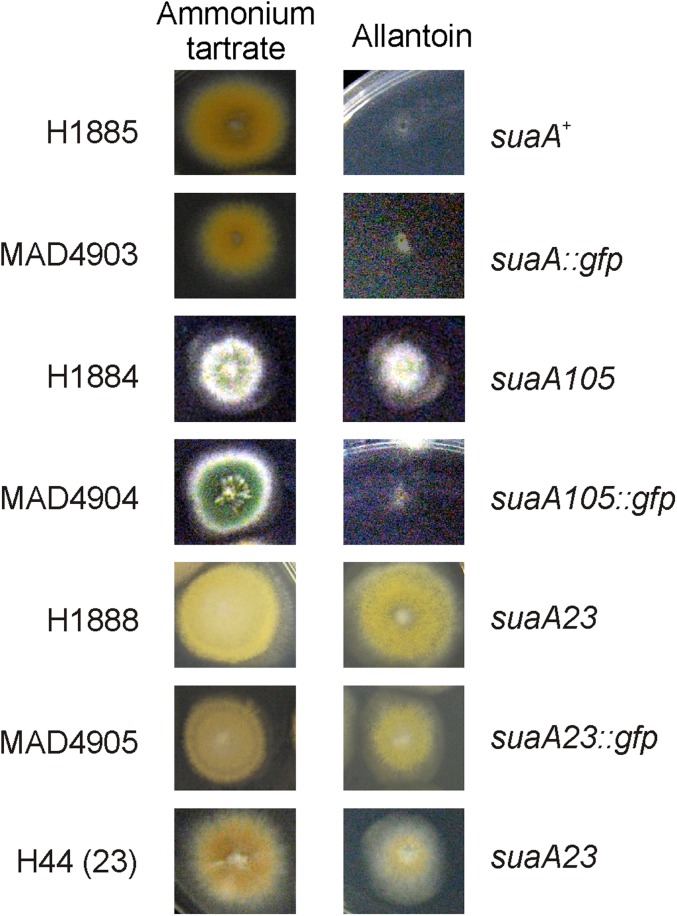
Growth tests of wild-type (*suaA^+^ alX4*) and suppressor strains (*suaA105* or *suaA23 alX4*) in comparison with transformants of an *alX4* containing strain with *suaA23* or *suaA105*::*gfp* constructs (see *Materials and Methods*). (Left) 1% glucose medium and 5 mM ammonium tartrate. (Right) 1% glucose medium with 0.6 mM allantoin as nitrogen source.

To investigate further the *suaA23* suppressor function, a construct containing the 5′UTR and *suaA23* coding region fused to the *gfp*/*riboB^Af^* cassette was used to transform strain H1888: *yA2 pantoB100 pabaA1*; *alX4 suaA23*; *riboB2* to prototrophy on riboflavin medium. DNA was sequenced from the transformants, which confirmed the presence of the *suaA23* mutation. The SuaA23-GFP fusion displayed a similar cellular distribution to SuaA-GFP (data not shown). The ability to suppress *alX4* was also confirmed for this mutant SuaA23-GFP fusion (Figure 4). SuaA^+^-GFP transformants did not show suppression of the *alX4* mutation but showed the presence of full wild-type SuaA function. These results demonstrate that the GFP tag does not interfere with SuaA function, and that *suaA23* affects functionality of eRF1 but not at the level of its cellular distribution.

**Figure 5:**
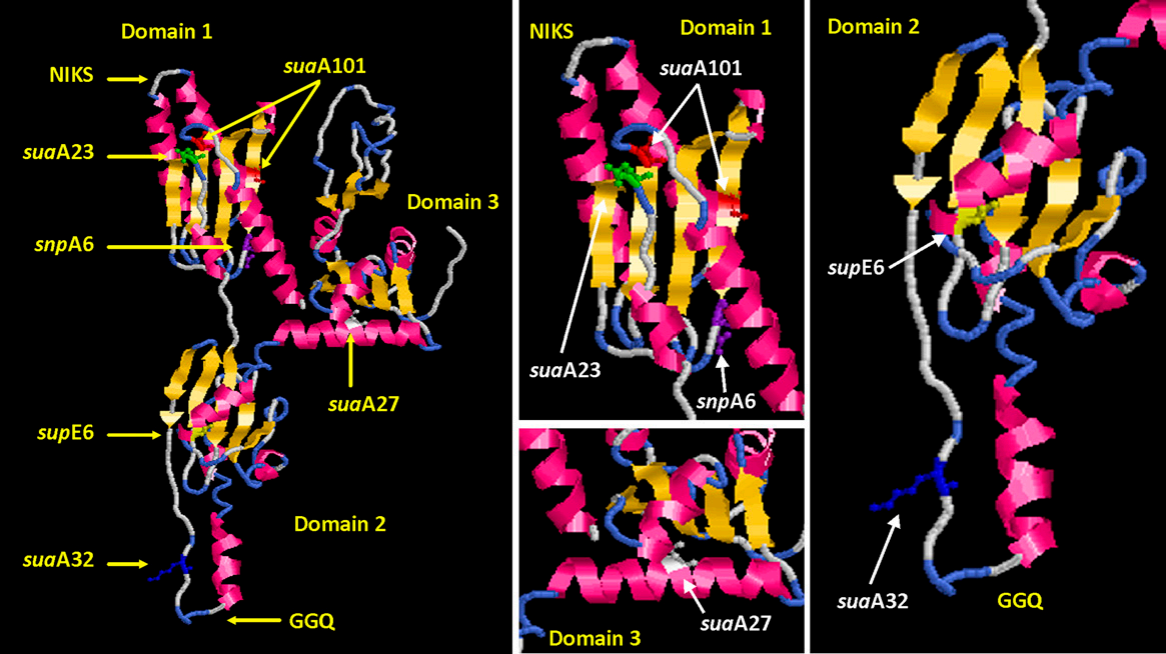
The three-dimensional structure of SuaA from *A. nidulans* has been modeled using the crystal structure of eRF1 from *H. sapiens* (PDB ID: ICT9) as a template through SWISS-MODEL. There is 77% identity between the amino acid sequences of eRF1 from *A. nidulans* and *H. sapiens*. The coordinates for the changes in *snpA1* and *supE6* were taken from Han *et al.* (2005) and Martinez *et al.* (2007). The amino acid changes have been highlighted in the following colors: green, *suaA23* (N131I); red, *suaA101 (*S35Y, N113K); purple, *snpA6* (E117K); yellow, *supE6* (G265S); blue, *suaA32* (G265S); and white, *suaA27* (I289S). (Left) All the *suaA* mutations on eRF1. (Right) Magnified images of all or parts of domains 1, 2, and 3 to show the positions of the amino acid changes more clearly.

The changes in the eRF1 have been located on the structure of the human eRF1 (Figure 5). eRF1 is a release factor that in combination with eRF3 is able to recognize the chain termination codons and elicit the release of the peptidyl tRNA from the ribosome. The structure of eRF1 mimics the structure of the tRNA and there are three recognizable domains: domain 1 (in human eRF1 amino acids 6 to 140), domain 2 (amino acids 144 to 276), and domain 3 (amino acids 279 to 417), which are analogous to the anticodon loop, the acceptor stem, and the T stem, respectively. The N-terminal domain 1 is responsible for recognition of the codon in the A-site of the ribosome and the most recent evidence suggests that the recognition is not by a linear sequence of amino acids but rather distinct motifs that are close together in the three-dimensional structure of the protein. The highly conserved motifs TASNIKS and YxCxxxF are part of the region that recognizes the stop codons (Bertram *et al.* 2000; Frolova *et al.* 2002; Bulygin *et al.* 2011). Domain 2 contains a conserved GGQ motif that interacts with the peptidyl transferase center in the ribosome and triggers peptidyl–tRNA hydrolysis (Frolova *et al.* 1999; Seit-Nebi *et al.* 2001) and is involved in stimulation of GTPase activity in eRF3 (Cheng *et al.* 2009). The C-terminal domain 3 is involved in binding eRF3, which has a ribosome-dependent GTPase activity: the hydrolysis of GTP is necessary for a conformational change in the eRF1 and eRF3 complex that allows the efficient release of the polypeptide (Alkalaeva *et al.* 2006; Fan-Minogue *et al.* 2008). Recent studies of *S. cerevisiae* suggest that the conformation of eRF1 might be different for recognition of UAA/UAG and UGA, with C124 and an associated epitope being important for UGA decoding (Fan-Minogue *et al.* 2008; Merritt *et al.* 2010).

**Figure 6:**
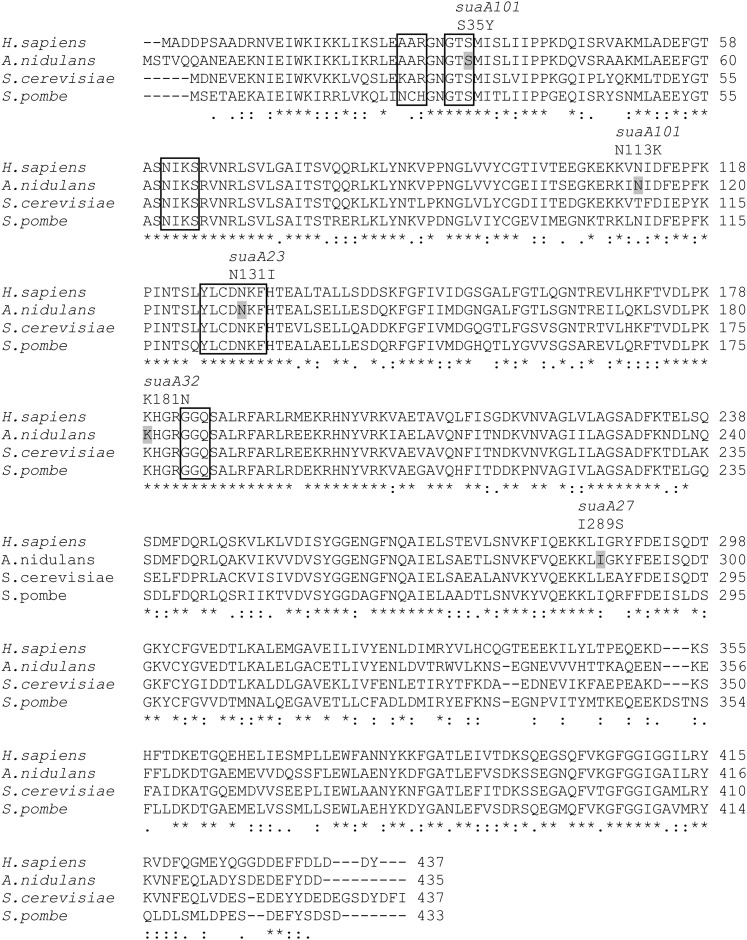
Multiple alignment of eRF1 sequences. ClustalW alignment of eRF1 from *H. sapiens* NP_004721, *S .cerevisiae* NP_009701.3, *S. pombe* NP_594680.1, and *A. nidulans* CBF77874 [AN8853 version 5 (aspergillusgenome .org)]. This version of the protein differs from that used by Han *et al.* (2005) because the most recent annotation includes further modifications of the transcript, which includes an intron in both the 3′ the 5′ non-coding regions and an additional intron at the 3′ end of the coding region, resulting in a protein that is 10 amino acids shorter at the C-terminus. *The positions where there is a fully conserved residue; (:) indicates that one of the following “strong” amino acids is conserved: STA NEQK, NHQK, NDEQ, MIL, MILF, HY, or FWY; and (.) denotes that one of the following “weaker” amino acids is conserved: CSA, ATV, SAG, STNK, STPA, SGND, SNDEQK, NDEQHK, NEQHRK, FVLIM, or HFY. Domain 1 of the protein using the *H. sapiens* numbering of the sequence is from amino acids 4 to 140; domain 2 is from amino acids 144 to 276; and domain 3 is from 279 to 417. Conserved functional motifs have been boxed and the mutational positions of the suppressors in *suaA* have been highlighted in the *A. nidulans* sequence, with the mutation indicated above the alignment.

*suaA23* involves a single amino acid change within the conserved YxCxxxF region [(N131I), YLCDNKF–YLCDIKF in the β-sheet domain 1], whereas *suaA101* involves two amino acid changes, an S35Y change and a change in the β-sheet region of domain 1 (N113K); both these suppressor strains have very similar specificities with regard to the suppressible alleles *alX4* (UAG), *areA600* (UAG), and *areA601* (UAA) (Sealy-Lewis 1987) (Figure 5 and Figure 6). They both suppress the null phenotype for *alX4* and *areA600* but not for *areA601*. The conserved NIKS region is involved in recognition of the first U of the codon. Recent studies using perfluorophenyl analogs of UAA and UAAA with human eRF1 have implicated the YxCxxxF motif as binding to purines in the second and third positions as well as a novel site 26-AAR-28. Position 28 is conserved as either arginine or lysine in a number of species and the positive side chains of these amino acids would interact with the negatively charged phosphates 3′ of adenosines in the third and fourth positions of the tetrapeptide (Fan-Minogue *et al.* 2008; Bulygin *et al.* 2011). Analogous studies using similar derivatives of guanine have shown that in addition to association with the YxCxxxF motif, the Thr-32 in the conserved 31-GTx-33 motif is the major target for cross-linking (Bulygin *et al.* 2010). Molecular modeling suggests that recognition of the UAG/UAA and UGA in stop codons is associated with a different conformation of eRF1 (Fan-Minogue *et al.* 2008). *suaA23* has a change within the conserved YxCxxxF region and can suppress the UAG mutations within *alX4* and *areA600* but is still able to terminate at the UAA mutation. An interpretation of these results is that the change in the YxCxxxF motif is able to discriminate between the UAG and UAA codon. In the case of *suaA101*, where there are two changes in the protein, it is not possible to deduce whether both of the changes are necessary for the phenotype, but the change S35Y produces a change in the conserved residue that was implicated as being important in human eRF1 for binding guanines (31-GTx-33 human numbering) and 33- GTS-35 in *A. nidulans* (Kryuchkova *et al.* 2013). The S35 residue has been changed and the *suaA101* strain is unable to terminate at the UAG codons within *alX4* and *areA600*, but it is able to terminate at the UAA codon. The alterations in eRF1 that lead to suppression reduce the efficiency of termination, but the protein must retain some function because there is only one copy of the termination factors eRF1 and eRF3 in *A. nidulans*, and termination must still be able to occur at normal stop codons. The context of the stop codon is very important. Where stop codons occur through mutation within a coding sequence that are not in a preferred context, these mutations will be more subject to read-through by the altered release factors. Suppression at these codons is dependent on the natural suppressor activity of normal tRNAs; which tRNA will be inserted is presumably a competition between these naturally occurring tRNAs and the release factors (Beier and Grimm 2001).

*suaA32* has a change in domain 2 (K181N) in the region of the GGQ domain that plays a role in the interaction of eRF1 with the peptidyl transferase center (Frolova *et al.* 2000) and is also close to the region implicated in the stimulation of the GTPase activity of eRF3 (Cheng *et al.* 2009). *suaA27* has a change in domain 3 in the β-sheet (I289S) (Figure 5 and Figure 6). In contrast to *suaA101*-containing and *suaA23*-containing strains, *suaA32 and suaA27* suppress *alX4*, *areA600*, and *areA601* mutations, thus having specificity for both UAG and UAA mutations. The *suaA32* and *suaA27* mutations are less likely to affect the discrimination between the three stop codons but would be more likely to generally reduce the efficiency of chain termination, and so they are able to suppress both the UAG and UAA mutations. The mutation in *suaA27* is in a conserved amino acid very close to the start of domain 3 that interacts with eRF3. The hydrophobic amino acids in *Schizosaccharomyces pombe* eRF1: Phe288, Ile291, Tyr298, Phe300, and Phe405 have been implicated in eRF3 interaction (amino acids within *A. nidulans* -5). From work in *S. cerevisiae*, it has been found that the structural changes within eRF1 can result in alterations in the level of eRF1, but the structural change itself can alter the read-through observed (Hatin *et al.* 2009; Merritt *et al.* 2010). We cannot distinguish between these alternatives in this study. It is evident, however, that mutations within eRF1 can lead to suppression of both UAG and UAA mutations. *suaA23*, *suaA105*, *suaA101*, and *suaA32* suppressor strains were crossed to the UGA containing *aldA67* mutant strain (Table 1) and there was no suppression of the *aldA* mutant phenotype at 37°, but at 25° some suppression was observed in the *suaA23 aldA67* (but not *suaA101 aldA67*), *suaA32 aldA67*, and *suaA105 aldA67* double mutant, showing that eRF1 acts on UGA as well as UAA and UAG mutations.

There were two previous publications in which suppressor mutations in eRF1 were described in *A. nidulans*. In the first article, Han *et al.* (2005) described a mutation in the pantothenyl transferase, *ngA1*, that resulted in a lack of pigmentation in the conidia and the hyphae that could be suppressed by a conditional mutation in a second gene, *snpA6*, at 37° and 42°, but not at 25°. The *snpA6* suppressor was shown to involve an E117K mutation in domain 1 eRF1 (Figure 5) of a UGA stop codon in *ngA1*. Martinez *et al.* (2007) described a suppressor, *supE7*, of two UAG mutations in the acetyl carnitine transferase carrier protein (*acuH13* and *acuH31* but not *acuH20*) that involved a change in domain 2 of eRF1 (G265S). It was proposed that this amino acid change might affect the conformational properties of the hinge region between domains 2 and 3, interfering with ribosome binding or peptidyl transferase activity (Figure 5). *supE7* also suppressed UAA and UGA mutations in *pal*^−^ strains (Martinez *et al.* 2007). The *supE7* suppressor mapped on chromosome III and had very similar properties to the *suaA* and *suaC* suppressors, but it was found by a cross with a *suaA101* to be unlinked to *suaA101*. Because the four *suaA* mutations have sequence changes in eRF1, all these mutations are in eRF1 like *supE7* (it seems likely that the cross data were incorrect but it has not been possible to repeat this cross because the *supE7* strain is no longer viable) and the *suaA* mutations can suppress all three classes of chain termination codon. We propose that *suaE7* and *snpA6* are reclassified as *suaA* mutations, because the *suaA* mutations were the first to be described and *suaA* is located on the genetic map. The only alteration that was found in the *suaA105* strain was in the region upstream of the coding region of the genes at −209 with respect to the start codon. This coincides with an alteration in the 5′ slice site, GT to AT, in the upstream ORF. We predicted that this would lead to a failure of splicing of the intron in the mutant that could affect the initiation of translation leading to lower levels of expression of eRF1. In RT-PCR of the wild-type and mutant RNA with a forward primer that split the first intron and a reverse primer that was after the second intron, only a PCR product was seen for the wild-type amplification, and sequencing of this product confirmed the position of the three introns as annotated. A PCR amplification where the forward primer was within the first intron and the reverse primer split the second intron in the coding sequence produced a product for the *suaA105* strain, but not for the wild-type strain, and sequencing of the PCR product confirmed the presence of an unspliced 5′ intron but correct splicing of the first two introns in the coding sequence (data not shown). Epitope (-GFP) tagging of the wild-type and mutant proteins resulted in reduced expression in the *suaA105* strain (Figure 3B). As described for other constructs, a genomic fragment containing *suaA105* mutation was fused to the *gfp/riboB^Af^* cassette and was used to transform strain H1884 (Table S1) to prototrophy on riboflavin medium. A feasible explanation for the effect of altered mRNA processing due to *suaA105* mutation is at the protein levels, because a 20% reduction is observed in the mutant background compared to wild-type. Lower levels of expression of eRF1 could lead to suppression by the natural tRNAs that are able to translate chain termination codons as sense, because they would be able to compete more efficiently for translation of the termination codons. However, the *suaA105*::*gfp* transformants did not show suppression of *alX4* (Figure 4), suggesting an interference of GFP in the functionality of this low-expressed but wild-type SuaA tagged protein.

### *suaC* codes for eRF3

The *suaC109* suppressor–containing strain is cold-sensitive (fails to grow at 25°). *suaC* maps on linkage group VII, shows linkage to *choA* (data not shown), and has a similar broad spectrum of suppression to the *suaA* suppressor strains. *suaC109* suppresses the UAG mutations in *alX4*, *alcR125*, and *areA600*, as well as the UAA mutation in *areA601* (Sealy-Lewis 1987) and *brlA17*, but not the UAA mutations in *brlA19* or *brlA4* or the UAG mutation in *brlA24* (Griffith *et al.* 1999). *suaC109* strains were crossed with UGA containing *pal*^−^ strains *palC143*, *palF15*, *palB7*, and *palB513* (mutations that affect pH regulation) in an attempt to establish whether *suaC109* could suppress UGA mutations, but the crosses were infertile. The location of *suaC* on the genetic map was in the region of eRF3, and so DNA extracted from the *suaC109* strain was sequenced and a single change T1186A (Y396N) was found in the sequence predicted to encode eRF3, AN2080. The sequence as annotated has one intron and encodes a protein of 708 amino acids. The eRF3 comprises two domains: the N and M regions (amino acids 1–253 in *S. cerevisiae*) and the C region (amino acids 254–685) that contain a GTPase fold (amino acids 254–479) and also interact with eRF1 (Kong *et al.* 2004). Crystallography studies between the eRF1 from *H. sapiens* and *S. pombe* and the C-terminus of the eRF3 from *S. pombe*, which lacks the GTPase domain, has shown that there is an interaction between the C-terminus of eRF1 and the C-terminus of eRF3, but there is additional evidence from small-angle X-ray scattering analysis that an interaction between domain 2 (R192 eRF1 *H. sapiens*) of eRF1 is required for stimulation of the GTP-binding and hydrolysis activities of eRF3 (Cheng *et al.* 2009).

There is considerable variability between eRF3s in the N and M domains in different eukaryotes, but the C-terminal domain is highly conserved. The *A. nidulans* eRF3 shows no similarity with the ERF3 from *H. sapiens* in the first 269 amino acids of the protein, but thereafter it has 53% identity. *S. cerevisiae* has no identity over the first 53 amino acids but has 48% identity thereafter, and *S. pombe* shows similarity over 99% of the protein with 49% identity. In *S. cerevisiae*, the N-terminal portion of the protein has been shown to be important for the cytoplasmically inherited prion determinant (PSI^+^), which when aggregated impairs termination and acts as a suppressor of nonsense mutations (Ter-Avanesyan *et al.* 1994). The N-terminal region contains four tandem repeats of the sequence, PQGGYQQYN, similar to mammalian prion repeats (Stansfield and Tuite 1994; Lindquist 1997). This sequence is missing from the sequence in *S. pombe* and *A. nidulans*, but *S. pombe* contains a region that is rich in repeats of APST (Ito *et al.* 1998) that, again, is not a feature of the *A. nidulans* sequence. Examination of the *A. nidulans* eRF3 for repeated elements using REPRO (http://www.ibi.vu.nl/programs/reprowww/) did not reveal any obvious repeats, but there was a preponderance of Glu, Ala, and Tyr amino acids between amino acids 53 to 156 in the N-terminus that would produce a very hydrophilic flexible region. In the meiotic analysis of many nonsense suppressor mutations in *A. nidulans*, there has never been any evidence of cytoplasmic inheritance as exhibited by the PSI^+^ strain in *S. cerevisiae* and, therefore, no evidence of prion formation (H. M. Sealy-Lewis, unpublished data).

**Figure 7:**
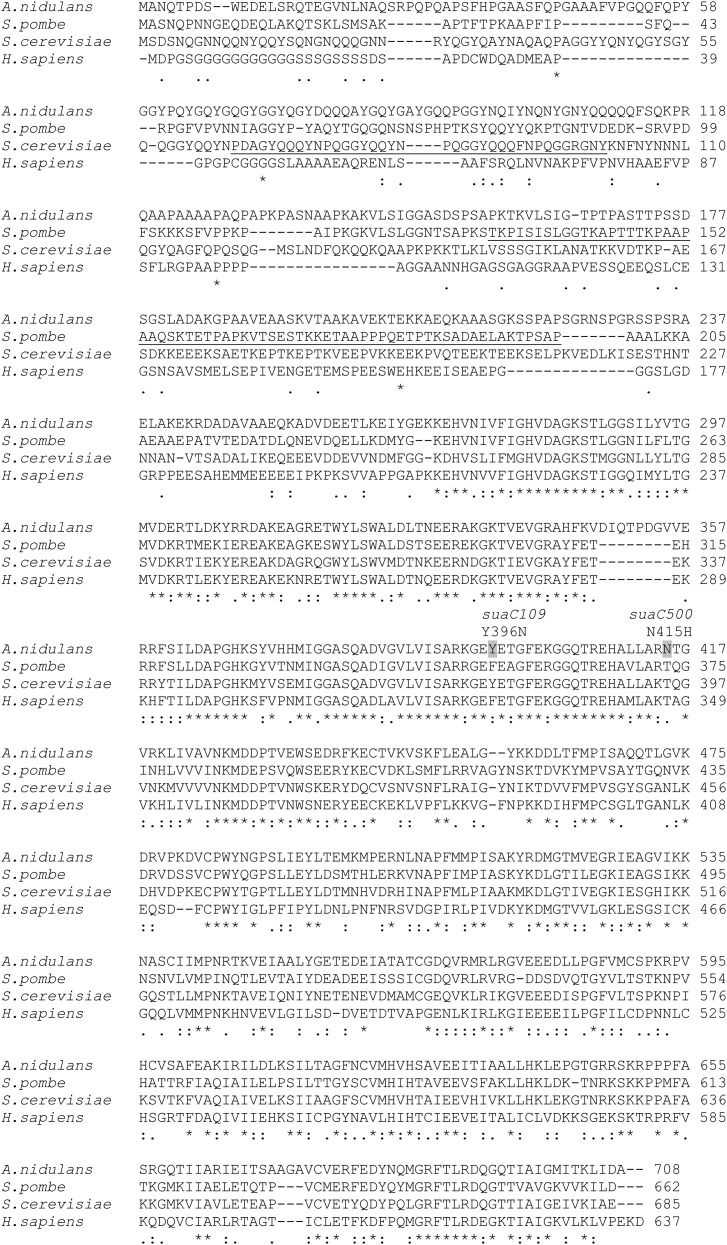
Multiple alignment of ERF3. Alignments using ClustalW are shown for *A. nidulans* XP_659684.1 (locus tag AN2080), *S. cerevisiae* AFD29160.1, *S. pombe* NP_588225, and *H. sapiens* ERF3A NP-002085.2. There are two genes in mammals that code for eRF3, eRF3A, and eRF3B, and they differ in their N-termini. We have used eRF3A for the alignment because the expression levels of this gene have been shown to control the formation of the termination complex (Chauvin *et al.* 2005). The explanation of conservations underneath the alignment is explained in Figure 6. Amino acid changes in the suppressor strains are indicated above the alignment with the amino acid that has change shaded. There are repeated sequences reported for both *S. cerevisiae* and *S. pombe*, and these are underlined in the respective sequences (see text for references).

**Figure 8:**
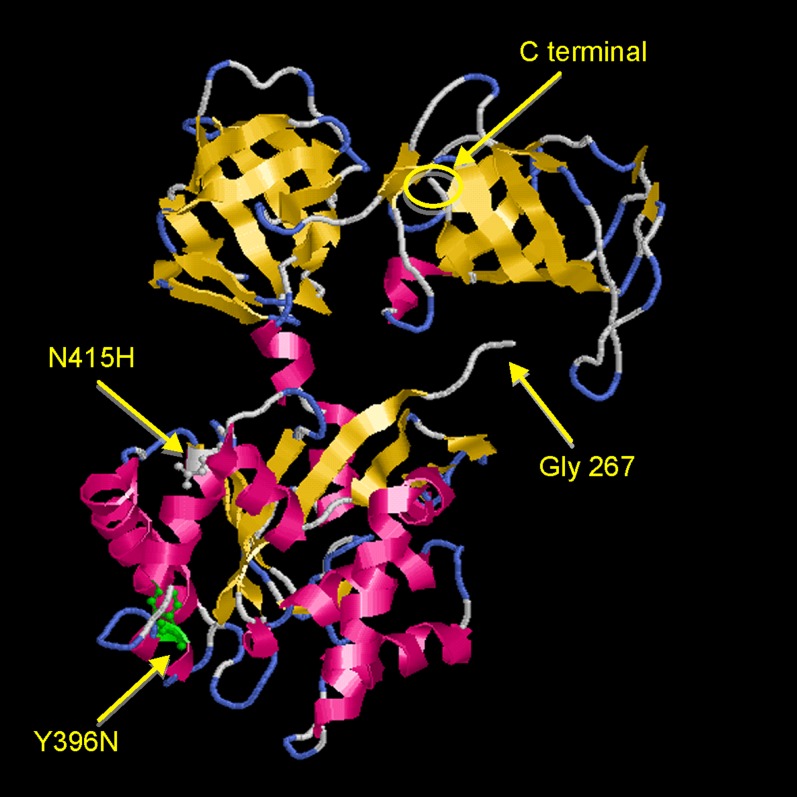
The three-dimensional structure of *suaC* from *A. nidulans* has been modeled using eRF3 from *S. pombe* (PDB ID:1R5B) as a template through SWISS-MODEL. The 1RB5 sequence used is 467 amino acids in length, covering residues 196 to 662 in *S. pombe*, and the sequence this corresponds to in *Aspergillus* is from amino acid 267 to the end of the sequence. *suaC109* (Y396N) and *suaC500* (N415H) are labeled as green and white, respectively.

The alteration in eRF3 in *suaC109* was in the C-terminus and has been located using the crystal structure of *S. pombe* for modeling (Table 2, Figure 7 and Figure 8). We also sequenced a mutant strain that was selected as a revertant of the cold-sensitive phenotype of *suaC109*. The *alX4* and *sB43* mutations were still suppressed to some extent in the revertant strain at both 37° and 25°. In an outcross between the *suaC109* revertant strain and the wild-type, there was no segregation of the cold-sensitive phenotype and we concluded that the mutations were allelic. Sequencing of the *suaC109* revertant strain revealed the change observed in *suaC109* together with a second change (N415H). The revertant of the *suaC109* phenotype has been renamed *suaC500*. Both changes are thus in the C-terminus (Table 2, Figure 7 and Figure 8). The revertant still had suppressor activity but was cold-insensitive. The cold sensitivity was thus a property of the altered eRF3 and not a consequence of a read-through product in an unrelated gene. Both *suaA* and *suaC* mutations result in a number of pleiotropic changes, which could result from read-through of proteins; in addition, it has been suggested for both eRF1 and eRF3 that they might have a translation-independent role, because eRF1 and the myosin-light chain have been shown to interact in *S. cerevisiae* and mutations in eRF1 can suppress defects in cytokinesis (Valouev *et al.* 2004). The N-terminal region of eRF3 in *S. cerevisiae* has also been shown to interact with other proteins such as Itt1p (a protein of unknown function) and Slap1, which is involved in cytoskeletal assembly; this suggests a translation-independent role for eRF3 (Bailleul *et al.* 1999; Urakov *et al.* 2001).

The reason for the cold sensitivity in the *suaC109* strain is unknown, but it could involve a failure to assemble with eRF1, GTP, or the ribosome at lower temperatures, as has been described for ribosome assembly in *E. coli* (Guthrie *et al.* 1969). This could be reversed by further changes within the eRF3 molecule. Also, in *S. cerevisiae* it has been shown that the GTPase domain of eRF3 interacts with Upf1p, which is involved in nonsense-mediated decay, and any changes that interfere with their interaction could also lead to nonsense suppression (Amrani *et al.* 2006; Ivanov *et al.* 2008). Genome-wide interaction studies have identified a large number of interactions between eRF3 and eRF1 and other proteins in *S. cerevisiae* (von der Haar and Tuite 2007), and there may be other proteins that interact with the GTPase domain of eRF3.

## Conclusion

The suppressor mutations in eRF1 are found in all three domains of the protein but in eRF3 they are confined to the conserved C-terminal domain, which is similar to the findings in *S. cerevisiae* (Merritt *et al.* 2010). The *suaA* and *suaC* suppressor strains were characterized by the fact that each mutant has a distinct but unique phenotype, and it is clear that the diverse interactions of eRF1 and eRF3 can lead to these properties. With regard to the *suaA* suppressors, they can result in temperature-sensitive phenotypes for some suppressed proteins but not others [*e.g.*, *suaA27* and *areA600* or *suaA101* and *amdS1005* result in a phenotype where growth is stronger at 25° than 37° (Sealy-Lewis 1987)]. This suggests that the same amino acid is not inserted at the stop codon for the different suppressor alleles and implies that the different eRF3 mutants compete differently with the natural suppressor tRNAs in the cell. The molecular details of how the release factors interact both with each other and with other factors on the ribosome should yield further insights into the process.

## Supplementary Material

Supporting Information
