# Supplementary material for: In Aspergillus nidulans the Suppressors suaA and suaC Code for Release Factors eRF1 and eRF3 and suaD Codes for a Glutamine tRNA
Source: G3 (Bethesda). 2014 Apr 9;4(6):1047–57. doi: 10.1534/g3.114.010702 (PMC4065248; doi:10.1534/g3.114.010702)
Supplement: Supporting Information [file supp_4_6_1047__index.html]

In Aspergillus nidulans the suppressors suaA and suaC code for release factors eRF1 and eRF3 and suaD codes for a glutamine tRNA — Supporting Information 

# In *Aspergillus nidulans* the Suppressors *suaA* and *suaC* Code for Release Factors eRF1 and eRF3 and *suaD* Codes for a Glutamine tRNA

## Supporting Information for Liu *et al.*, 2014

**Files in this Data Supplement:**

- Supporting Information - Tables S1-S2 (PDF, 157 KB)
- Table S1 - Strains used. (PDF, 79 KB)
- Table S2 - Primers used in the study. (PDF, 73 KB)
