# Supplementary material for: In Aspergillus nidulans the Suppressors suaA and suaC Code for Release Factors eRF1 and eRF3 and suaD Codes for a Glutamine tRNA
Source: G3 (Bethesda). 2014 Apr 9;4(6):1047–57. doi: 10.1534/g3.114.010702 (PMC4065248; doi:10.1534/g3.114.010702)
Supplement: Supporting Information [file supp_g3.114.010702_010702SI.pdf]

G3·Genes | Genomes | Genetics

In *Aspergillus nidulans* the suppressors *suaA* and *suaC* code for release factors eRF1 and eRF3 and *suaD* codes for a glutamine tRNA

Wen Liu[*], Laura Mellado[§], Eduardo A. Espeso[§] and Heather M. Sealy-Lewis[*]

[*]Department of Biological Sciences
University of Hull
Hull HU6 7RX
United Kingdom

[§]Department of Cellular and Molecular Biology
Centro de Investigaciones Biológicas (CSIC)
Ramiro de Maeztu, 9, 28040
Madrid
Spain

Corresponding author:
Heather M. Sealy-Lewis
Department of Biological Sciences
University of Hull
Hull HU6 7RX
United Kingdom
4401482 465970
h.m.sealy-lewis@hull.ac.uk

DOI: 10.1534/g3.114.010702

Table S1 Strains used

Strains[a]	Genotype	Reference
H44	*pabaA1: alX4; alcR125; niaD500 fwA1*	Roberts *et al*.1979
H44 (32)	*pabaA1: alX4; alcR125; niaD500 fwA1 suaA32*	(Sealy-Lewis 1987)
H44 (23)	*pabaA1: alX4; alcR125; niaD500 fwA1; suaA23*	(Sealy-Lewis 1987)
H44 (27)	*pabaA1: alX4; alcR125; niaD500 fwA1; suaA27*	(Sealy-Lewis 1987)
H3	*yA2: alX4 suaA105; pantoB100*	(Roberts *et al*. 1979)
H103	*alX4 suaA101; pantoB100; fwA1 niaD500;*	(Roberts *et al*. 1979)
H9	*pabaA1; alX4 suaB111; sB43; fwA1*	(Roberts *et al*. 1979)
H7	*pabaA1; alX4; sB43; fwA1; suaC109*	(Roberts *et al*. 1979)
H7(16.1)	*pabaA1; alX4; sB43; fwA1; suaC109 suaC500*	This study
H2	*yA2; alX4; pyroA4; sB43; suaD103*	(Roberts *et al*. 1979)
H16	*pabaA1; alX4; sB43; suaD108; fwA1*	(Roberts *et al*. 1979)
H1859	*biA1; alX4; aldA67*	This study
H1885	*yA2 pantoB100; sB43; aldA67 riboB2*	This study
H1884	*alX4 suaA105; riboB2*	This study
H1888	*pabaA1;yA2 pantoB100; alX4; riboB2; suaA23*	This study
MAD2733	*pabaA1; argB2; ∆nkuA::argB*	(Markina-Iñarrairaegui *et al*. 2011)
MAD1427	*pyrG89, pabaB22; argB2; ∆nkuA::argB; riboB2*	(Markina-Iñarrairaegui *et al*. 2011)
MAD4903	*yA2 pantoB100; sB43; aldA67 riboB2 suaA::gfp::*$riboB^{Af}$	This study
MAD4904	*alX4 suaA105::gfp::*$riboB^{Af}$ *; riboB2*	This study
MAD4905	*pabaA1;yA2 pantoB100; alX4; riboB2; suaA23::gfp::*$riboB^{Af}$	This study
wild type	*pabaA1*	
2047	*pabaA1; palB7*	(Peñas *et al*. 2007)
2790	*areA5; inoB; glrA1; palB513*	(Peñas *et al*. 2007)
1138	*biA1;* $areA^{r}$*18; palC143*	(Tilburn *et al*. 2005)
2225	*glrA1; pantoB100 palF15*	(Herranz *et al*. 2005)

[a] All strains used were *veA1*

Reference

Markina-Iñarrairaegui, A., and O. Etxebeste, Herrero-Garcia, E., L. Araujo-Bazan, J. Fernandez-Martinez et al., 2011 Nuclear transporters in a multinucleated organism: functional and localization analyses in *Aspergillus nidulans*. Mol. Biol. Cell 22: 3874–3886.

Table S2 Primers used in the study

Primer name	Primers for amplification and sequencing of eRF1
eRF1F1	ATGATGGGACTTTGGGCTAA
eRF1R1	TTCGCTGCTCTGGATACTTGA
eRF1F2	CGTCAACTCGCGATATTGACA
eRF1R2	ATACCGGCGACATTGACTTT
eRF1F3	TCCAAAAGCTTAGCGTCGAT
eRF1R3	CCGTCAAAAGATCCGTGTTT
	Primers for amplification of eRF1(SuaA)-GFP tagging cassettes
suaA1	TCCGGACCTGGTACCAGCCTTCAG
suaA2	GGCAACCTCGGTGACTACTTTACGG
suaA3	CCGTAAAGTAGTCACCGAGGTTGCCGGAGCTGGTGCAGGCGCTGGAGC
suaA4	AGGCTCCATGGTGTTATTCGCATGAAG
suaA5	ATGATCTACAGGTGATGTTGAGACGGG
suaA6	CTTCATGCGAATAACACCATGGAGCCTCTGTCTGAGAGGAGGCACTGATG
	Primer used in sequencing of *suaA105* and *suaA23* mutations
eRF1 up	GTCTCAAGAACCAGGTGG
	Primers for amplification of eRF3
eRF3F1	GTCTTGTTCGAGCGGTTG
eRF3R1	CTTCGCATCCCTCCTCTA
eRF3F2	GGCAGACGTTGATGAGGA
eRF3R2	GGGTCCCTGGGATACCTT
	Primers for amplification of *suaD*
suaDF1	AAGACCAGGAGTGGAGCG
suaDR1	AGGCGAAGACTGTGGGAG
	Primers for amplification of *alX4*
alX4F1	TGATTCCTGCTCGGTCTC
alX4R1	GAGGTAATGGTAGCAGGTTT
alX4F2	TCCCTCAGAATACGAGCTTT
alX4R2	TGAAGAGAAGTGTAGGATGC
	Primers for RT-PCR amplification to include UTR eRF1
eRF1UTRF5	AAAGTGACCAGCCCTCAAGAA
eRF1UTR4	TTTGAGACGTTGCTGGGTAGA
eRF1UTRF2	TCCTTACATGGCCCGAAA
eRF1UTRR1	AGTGCCAAATTCTTCCAGCCA
F1(Bt3)	GCTCCGGTGTTTACAATGG
R1(BT	AGTTGTTACCAGCGGAG
