# Supplementary material for: In Aspergillus nidulans the Suppressors suaA and suaC Code for Release Factors eRF1 and eRF3 and suaD Codes for a Glutamine tRNA
Source: G3 (Bethesda). 2014 Apr 9;4(6):1047–57. doi: 10.1534/g3.114.010702 (PMC4065248; doi:10.1534/g3.114.010702)
Supplement: Supporting Information [file supp_g3.114.010702_TableS1.pdf]

Table S1 Strains used

Strains[a]	Genotype	Reference
H44	*pabaA1: alX4; alcR125; niaD500 fwA1*	Roberts *et al*.1979
H44 (32)	*pabaA1: alX4; alcR125; niaD500 fwA1 suaA32*	(Sealy-Lewis 1987)
H44 (23)	*pabaA1: alX4; alcR125; niaD500 fwA1; suaA23*	(Sealy-Lewis 1987)
H44 (27)	*pabaA1: alX4; alcR125; niaD500 fwA1; suaA27*	(Sealy-Lewis 1987)
H3	*yA2: alX4 suaA105; pantoB100*	(Roberts *et al*. 1979)
H103	*alX4 suaA101; pantoB100; fwA1 niaD500;*	(Roberts *et al*. 1979)
H9	*pabaA1; alX4 suaB111; sB43; fwA1*	(Roberts *et al*. 1979)
H7	*pabaA1; alX4; sB43; fwA1; suaC109*	(Roberts *et al*. 1979)
H7(16.1)	*pabaA1; alX4; sB43; fwA1; suaC109 suaC500*	This study
H2	*yA2; alX4; pyroA4; sB43; suaD103*	(Roberts *et al*. 1979)
H16	*pabaA1; alX4; sB43; suaD108; fwA1*	(Roberts *et al*. 1979)
H1859	*biA1; alX4; aldA67*	This study
H1885	*yA2 pantoB100; sB43; aldA67 riboB2*	This study
H1884	*alX4 suaA105; riboB2*	This study
H1888	*pabaA1;yA2 pantoB100; alX4; riboB2; suaA23*	This study
MAD2733	*pabaA1; argB2; ΔnkuA::argB*	(Markina-Iñarrairaegui *et al*. 2011)
MAD1427	*pyrG89, pabaB22; argB2; ΔnkuA::argB; riboB2*	(Markina-Iñarrairaegui *et al*. 2011)
MAD4903	*yA2 pantoB100; sB43; aldA67 riboB2 suaA::gfp::riboB^{Af}*	This study
MAD4904	*alX4 suaA105::gfp::riboB^{Af} ; riboB2*	This study
MAD4905	*pabaA1;yA2 pantoB100; alX4; riboB2; suaA23::gfp::riboB^{Af}*	This study
wild type	*pabaA1*	
2047	*pabaA1; palB7*	(Peñas *et al*. 2007)
2790	*areA5; inoB; glrA1; palB513*	(Peñas *et al*. 2007)
1138	*biA1; areA^{r}18; palC143*	(Tilburn *et al*. 2005)
2225	*glrA1; pantoB100 palF15*	(Herranz *et al*. 2005)

[a] All strains used were *veA1*

Reference

Markina-Iñarrairaegui, A., and O. Etxebeste, Herrero-Garcia, E., L. Araujo-Bazan, J. Fernandez-Martinez et al., 2011 Nuclear transporters in a multinucleated organism: functional and localization analyses in *Aspergillus nidulans*. Mol. Biol. Cell 22: 3874–3886.
