# Supplementary material for: In Aspergillus nidulans the Suppressors suaA and suaC Code for Release Factors eRF1 and eRF3 and suaD Codes for a Glutamine tRNA
Source: G3 (Bethesda). 2014 Apr 9;4(6):1047–57. doi: 10.1534/g3.114.010702 (PMC4065248; doi:10.1534/g3.114.010702)
Supplement: Supporting Information [file supp_g3.114.010702_TableS2.pdf]

Table S2 Primers used in the study

Primer name	Primers for amplification and sequencing of eRF1
eRF1F1	ATGATGGGACTTTGGGCTAA
eRF1R1	TTCGCTGCTCTGGATACTTGA
eRF1F2	CGTCAACTCGCGATATTGACA
eRF1R2	ATACCGGCGACATTGACTTT
eRF1F3	TCCAAAAGCTTAGCGTCGAT
eRF1R3	CCGTCAAAAGATCCGTGTTT
	Primers for amplification of eRF1(SuaA)-GFP tagging cassettes
suaA1	TCCGGACCTGGTACCAGCCTTCAG
suaA2	GGCAACCTCGGTGACTACTTTACGG
suaA3	CCGTAAAGTAGTCACCGAGGTTGCCGGAGCTGGTGCAGGCGCTGGAGC
suaA4	AGGCTCCATGGTGTTATTCGCATGAAG
suaA5	ATGATCTACAGGTGATGTTGAGACGGG
suaA6	CTTCATGCGAATAACACCATGGAGCCTCTGTCTGAGAGGAGGCACTGATG
	Primer used in sequencing of *suaA105* and *suaA23* mutations
eRF1 up	GTCTCAAGAACCAGGTGG
	Primers for amplification of eRF3
eRF3F1	GTCTTGTTCGAGCGGTTG
eRF3R1	CTTCGCATCCCTCCTCTA
eRF3F2	GGCAGACGTTGATGAGGA
eRF3R2	GGGTCCCTGGGATACCTT
	Primers for amplification of *suaD*
suaDF1	AAGACCAGGAGTGGAGCG
suaDR1	AGGCGAAGACTGTGGGAG
	Primers for amplification of *alX4*
alX4F1	TGATTCCTGCTCGGTCTC
alX4R1	GAGGTAATGGTAGCAGGTTT
alX4F2	TCCCTCAGAATACGAGCTTT
alX4R2	TGAAGAGAAGTGTAGGATGC
	Primers for RT-PCR amplification to include UTR eRF1
eRF1UTRF5	AAAGTGACCAGCCCTCAAGAA
eRF1UTR4	TTTGAGACGTTGCTGGGTAGA
eRF1UTRF2	TCCTTACATGGCCCGAAA
eRF1UTRR1	AGTGCCAAATTCTTCCAGCCA
F1(Bt3)	GCTCCGGTGTTTACAATGG
R1(BT	AGTTGTTACCAGCGGAG
